# Lactic acid fermentation as a tool to enhance the functional features of *Echinacea* spp

**DOI:** 10.1186/1475-2859-12-44

**Published:** 2013-05-04

**Authors:** Carlo Giuseppe Rizzello, Rossana Coda, Davinia Sánchez Macías, Daniela Pinto, Barbara Marzani, Pasquale Filannino, Giammaria Giuliani, Vito Michele Paradiso, Raffaella Di Cagno, Marco Gobbetti

**Affiliations:** 1 Dipartimento di Scienze del Suolo, della Pianta e degli Alimenti, University of Bari, Via G. Amendola 165/a, 70126 Bari, Italy; 2 Agriculture and Livestock Engineering Faculty, Universidad Estatal del Sur de Manabí, Jipijapa 130650 Manabí, Ecuador; 3 Giuliani S.p.A, Milano 20129 Italy

## Abstract

**Background:**

Extracts and products (roots and/or aerial parts) from *Echinacea* ssp. represent a profitable market sector for herbal medicines thanks to different functional features. Alkamides and polyacetylenes, phenols like caffeic acid and its derivatives, polysaccharides and glycoproteins are the main bioactive compounds of *Echinacea* spp. This study aimed at investigating the capacity of selected lactic acid bacteria to enhance the antimicrobial, antioxidant and immune-modulatory features of *E. purpurea* with the prospect of its application as functional food, dietary supplement or pharmaceutical preparation.

**Results:**

*Echinacea purpurea* suspension (5%, wt/vol) in distilled water, containing 0.4% (wt/vol) yeast extract, was fermented with *Lactobacillus plantarum* POM1, 1MR20 or C2, previously selected from plant materials. Chemically acidified suspension, without bacterial inoculum, was used as the control to investigate functional features. *Echinacea* suspension fermented with *Lb. plantarum* C2 exhibited a marked antimicrobial activity towards Gram-positive and -negative bacteria. Compared to control, the water-soluble extract from *Echinacea* suspension fermented with *Lactobacillus plantarum* 1MR20 showed twice time higher radical scavenging activity on DPPH. Almost the same was found for the inhibition of oleic acid peroxidation. The methanol extract from *Echinacea* suspension had inherent antioxidant features but the activity of extract from the sample fermented with strain 1MR20 was the highest. The antioxidant activities were confirmed on Balb 3T3 mouse fibroblasts. *Lactobacillus plantarum* C2 and 1MR20 were used in association to ferment *Echinacea* suspension, and the water-soluble extract was subjected to ultra-filtration and purification through RP-FPLC. The antioxidant activity was distributed in a large number of fractions and proportional to the peptide concentration. The antimicrobial activity was detected only in one fraction, further subjected to nano-LC-ESI-MS/MS. A mixture of eight peptides was identified, corresponding to fragments of plantaricins PlnH or PlnG. Treatments with fermented *Echinacea* suspension exerted immune-modulatory effects on Caco-2 cells. The fermentation with *Lb. plantarum* 1MR20 or with the association between strains C2 and 1MR20 had the highest effect on the expression of *TNF*-α gene.

**Conclusions:**

*E. purpurea* subjected to lactic acid fermentation could be suitable for novel applications as functional food dietary supplements or pharmaceutical preparations.

## Background

The genus *Echinacea* belongs to the *Asteraceae* family and comprises a group of perennial prairie wildflowers, which are native to central grasslands of North America. The cultivation is mainly extended throughout United States, Canada and Europe (especially Germany). *Echinacea angustifolia* DC, *Echinacea pallida* (Nutt.) and, especially, *Echinacea purpurea* (L.) Moench [1] are the species more widespread [2]. High costs and scarce standardization of the spontaneous collections [3] had favored the expansion of the cultivation of *Echinacea* ssp. Extracts and products (roots and/or aerial parts) from the whole plant represent a profitable market sector for herbal medicines in North America and Europe [2]. The cultivation of *Echinacea* spp. in Italy corresponds to ca. 15 - 20 ha, with a production of ca. 9 - 12 tons of dried roots per year, but the demand is at least twice [3].

Alkamides and polyacetylenes [4], phenols like caffeic acid and its derivatives (caftaric, chlorogenic and cichoric acids, and cynarin and echinacoside) [5], polysaccharides [6] and glycoproteins [7] are the main bioactive compounds of *Echinacea* spp. Since this large variety of inherent functional compounds, the assignment of functional features to a defined class of chemical compounds is not always possible. Antioxidant, anti-inflammatory, antimicrobial and immune-modulatory activities are the main functional features of this herbal medicine. Alkamides have medicinal efficacy [8,9] and, more in general, extracts of *E. angustifolia* show therapeutic activity on adults who practice enduring sports [10]. Cichoric acid possesses immune-stimulatory [11], antiyaluronidase [12] and antiviral [5] activities, promotes the *in vitro* and *in vivo* phagocyte activity, protects collagen from free radical induced degradation [13], and inhibits HIV-1 integrase and replication [14,15]. Echinacoside protects the collagen from reactive oxygen species [13], and possesses antioxidant [16], anti-inflammatory and cicatrizing activities [17]. Preparations of *E. purpurea* are used for treatments of respiratory, urinary, viral and cutaneous infections, which are mainly related to deficiencies of the immune response [17-19]. Extracts from the same species had bactericidal activity towards *Staphylococcus* sp., and killed WEHI 164 tumor cells and the parasite *Leishmania enriettii* or *Candida albicans*[20]. Under near UV irradiation (phototoxicity), hexane extracts of *Echinacea* spp. inhibited the growth of numerous yeasts such as *Saccharomyces cerevisiae*, *Candida shehata*, *Candida kefyr*, *C. albicans*, *Candida steatulytica* and *Candida tropicalis*[21]. Extracts from *E*. *purpurea* protected immune-suppressed mice towards systemic infections by *Listeria monocytogenes* and *C. albicans*[22]. *In vivo* studies on mice, rat and humans showed the absence of toxicity of extracts of *Echinacea* spp., even when they were administered intravenously at high doses [23].

Based on the above description, the need to better define and, if possible, to enhance the activity of bioactive compounds emerges as a priority to get standardized market products (e.g., tablets, capsules and hydro-alcoholic extracts) for well-known or novel applications [1]. In compliance with this aim, a biotechnology approach, which is based on the use of plant cell cultures of *E. angustifolia*, was promoted by FAO, as an alternative method to produce secondary metabolites for food and pharmaceutical applications [24]. On the other hand, lactic acid fermentation of *Echinacea* spp. may also have the potential to standardize the functional features of the raw matrix and it may increase the bioavailability of certain compounds and/or allow the synthesis of novel substances to be used for functional preparations. Overall, the synthesis of bioactive metabolites having therapeutic properties is largely documented during lactic fermentation of plant materials [25-27].

To the best of our knowledge, no studies have already considered the use of lactic acid fermentation to enhance the functional features of *Echinacea* spp. This study aimed at investigating the capacity of selected lactic acid bacteria to enhance the antimicrobial, antioxidant and immune-modulatory features of *E. purpurea* with the prospect of its application as functional food, dietary supplement or pharmaceutical preparation.

## Results

### Echinacea fermentation

Echinacea powder suspension (ES) (5%, wt/vol) in distilled water or grape must (1% of total carbohydrates) allowed a very poor growth of *Lactobacillus plantarum* POM1, 1MR20 and C2. When yeast extract (0.4%, wt/vol) was added to suspension in distilled water, the cell density of the above strains increased from ca. 1 × 10^8^ CFU/ml to 2.2 ± 0.1 - 4.7 ± 0.3 × 10^9^ CFU/ml. All further experiments referred to these optimized culture conditions. *Lb. plantarum* 1MR20, C2 and POM1 caused an almost similar acidification. After 24 of fermentation, the values of pH of ES decreased from 5.21 ± 0.22 (control) to 4.35 ± 0.21, 4.18 ± 0.18, and 4.27 ± 0.13, respectively for *Lb. plantarum* 1MR20, C2 and POM1.

### Antimicrobial and antioxidant activities

The antimicrobial and antioxidant activities of control (ES-CT) and fermented ES were assayed *in vitro*. The inhibition of various indicator strains is shown in Table 1. No antimicrobial activity was found for ES-CT. ES fermented with *Lb. plantarum* 1MR20-ES showed a slight inhibition, only towards *Bacillus megaterium* F6. On the contrary, ES fermented with *Lb. plantarum* C2 and POM1 had rather wide and common spectrum of activity. The only exception were *Enterococcus durans* DSM20633, only inhibited by *Lb. plantarum* C2, and *Lactobacillus sakei* SAL1, *Candida krusei* DSM 3433 and *Penicillium roqueforti* DPPMA1, which were not affected. The antimicrobial activity of ES fermented by *Lb. plantarum* C2 was the most intense.

**Table 1:** Inhibitory spectrum of the Echinacea suspension (ES)

**Microorganism**	**ES-CT**	**1MR20-ES**	**C2-ES**	**POM1-ES**	**1MR20/C2-ES**
*Escherichia coli* DSM 30083	**-**	**-**	**++**	**+**	**++**
*Enterococcus durans* DSM 20633	**-**	**-**	**++**	**-**	**++**
*Yersinia enterocolitica* DSM 4780	**-**	**-**	**+++**	**++**	**+++**
*Bacillus megaterium* F6	**-**	**+**	**++++**	**+++**	**++++**
*Enterobacter aerogenes* DSM 30053	**-**	**-**	**+++**	**+**	**+++**
*Weissella confusa* DSM 20196	**-**	**-**	**+++**	**++**	**+++**
*Leuconostoc lactis* DSM 20202	**-**	**-**	**+++**	**+++**	**+++**
*Lactobacillus sakei* SAL1	**-**	**-**	**-**	**-**	**-**
*Propionibacterium jensenii* DSM 20535	**-**	**-**	**++**	**+**	**++**
*Candida krusei* DSM 3433	**-**	**-**	**-**	**-**	**-**
*Penicillium roqueforti* DPPMA1	**-**	**-**	**-**	**-**	**-**

Inhibitory activity was scored as follows: **-**: no inhibition; **+**: inhibition zone diameter 0.5-1 mm; **++**: inhibition zone diameter 1-2.5 mm; **+++**: inhibition zone diameter 2.5-3.5 mm; ++++: inhibition zone diameter >3.5 mm.

ES was fermented for 24 h at 30°C with *Lactobacillus plantarum* 1MR20 (1MR20-ES), C2 (C2-ES) and POM1 (POM1-ES), and with the association between strains 1MR20 and C2 (1MR20/C2-ES), as determined by agar diffusion assay. The chemically acidified ES, without bacterial inoculum, and incubated for 24 h at 30°C (ES-CT), was the control.

First, the antioxidant activity of fermented ES was assayed as radical scavenging activity on 1,1-diphenyl-2-picrylhydrazyl (DPPH) radical and as inhibition of oleic acid peroxidation. The analyses were carried out using both water-soluble (WSE) and methanol (ME) extracts. During radical scavenging assay, the colored stable DPPH radical is reduced to non-radical DPPH-H, when in the presence of an antioxidant or a hydrogen donor. DPPH radical without antioxidants was stable over the time. Under the assay conditions, the 100% of activity corresponds to the complete scavenging of DPPH radical (50 μM final concentration) after 10 min of incubation with the antioxidant compounds. According to previous studies [27,28], the color intensity of DPPH˙ showed a logarithmic decline when it was in the presence of butylated hydroxytoluene (BHT). The activity of WSE was lower than BHT, which was used as the positive control (Table 2). WSE from ES-CT had a radical scavenging activity towards the stable radical DPPH of 21.0 ± 0.2%. Fermentation significantly (*P*<0.05) increased the radical scavenging activity of all WSE. The increase was slight for ES fermented with *Lb. plantarum* C2 and POM1, but almost twice when strain 1MR20 was used. Compared to WSE, the ME from ES-CT had a higher radical scavenging activity. The antioxidant activities of ME from ES fermented with *Lb. plantarum* C2 and POM1 were slightly lower compared to ES-CT. On the contrary, the radical scavenging activity of ME from the ES fermented with strain 1MR20 was higher than that found for ES-CT and not significantly (*P*>0.05) different with respect to BHT. Lipid peroxidation is thought to proceed via radical mediated abstraction of hydrogen atoms from methylene carbons in polyunsaturated fatty acids [29]. The antioxidant activity of WSE from fermented and non-fermented ES was slightly lower than that of BHT used as the positive control. Compared to ES-CT, no significant (*P*>0.05) differences were found for the ES fermented with *Lb. plantarum* 1MR20. The values for the ES fermented with the other two strains were significantly (*P*<0.05) lower. The inhibition of the linoleic acid peroxidation by ME was similar to that of α-tocopherol and lower than that of BHT. No significant (*P*>0.05) differences were found among ES-CT and fermented ES.

**Table 2:** **Radical scavenging activity towards DPPH**^**• **^**and linoleic acid peroxidation inhibitory activity**

	**WSE**		**ME**	
	***DPPH radical scavenging activity***	***Lipid peroxidation inhibitory activity***	***DPPH radical scavenging activity***	***Lipid peroxidation inhibitory activity***
**BHT**	63.7 ± 0.5^d^	93.8 ± 0.3^d^	78.5 ± 0.3^c^	93.8 ± 0.2^c^
**α -tocopherol**	nd	91.9 ± 0.3^b^	nd	91.9 ± 0.2^a^
**ES-CT**	21.0 ± 0.2^a^	92.9 ± 0.2^c^	74.8 ± 0.4^b^	91.1 ± 0.3^b^
**1MR20-ES**	41.5 ± 0.4^c^	92.7 ± 0.3^c^	79.1 ± 0.2^c^	90.8 ± 0.3^b^
**C2-ES**	25.6 ± 0.4^b^	92.0 ± 0.4^b^	72.3 ± 0.3^a^	91.4 ± 0.1^b^
**POM1-ES**	24.6 ± 0.3^b^	90.5 ± 0.2^a^	75.3 ± 0.2^b^	90.2 ± 0.2^a^
**1MR20/C2-ES**	41.7 ± 0.2^c^	92.9 ± 0.1^c^	79.3 ± 0.1^c^	91.0 ± 0.2^b^

Data are the mean of three independent analyses.

^a-d^ Values in the same row with different superscript letters differ significantly (*P*<0.05).

Antioxidant activity of the water-soluble (WSE) and methanol (ME) extracts of Echinacea suspension (ES) fermented for 24 h at 30°C with *Lactobacillus plantarum* 1MR20 (1MR20-ES), C2 (C2-ES) and POM1 (POM1-ES), and with the association between strains 1MR20 and C2 (1MR20/C2-ES). The chemically acidified ES, without bacterial inoculum, and incubated for 24 h at 30°C (ES-CT), was the control. Butylatedhydroxytoluene (BHT) and α–tocopherol were used as positive controls.

**Figure 1:**
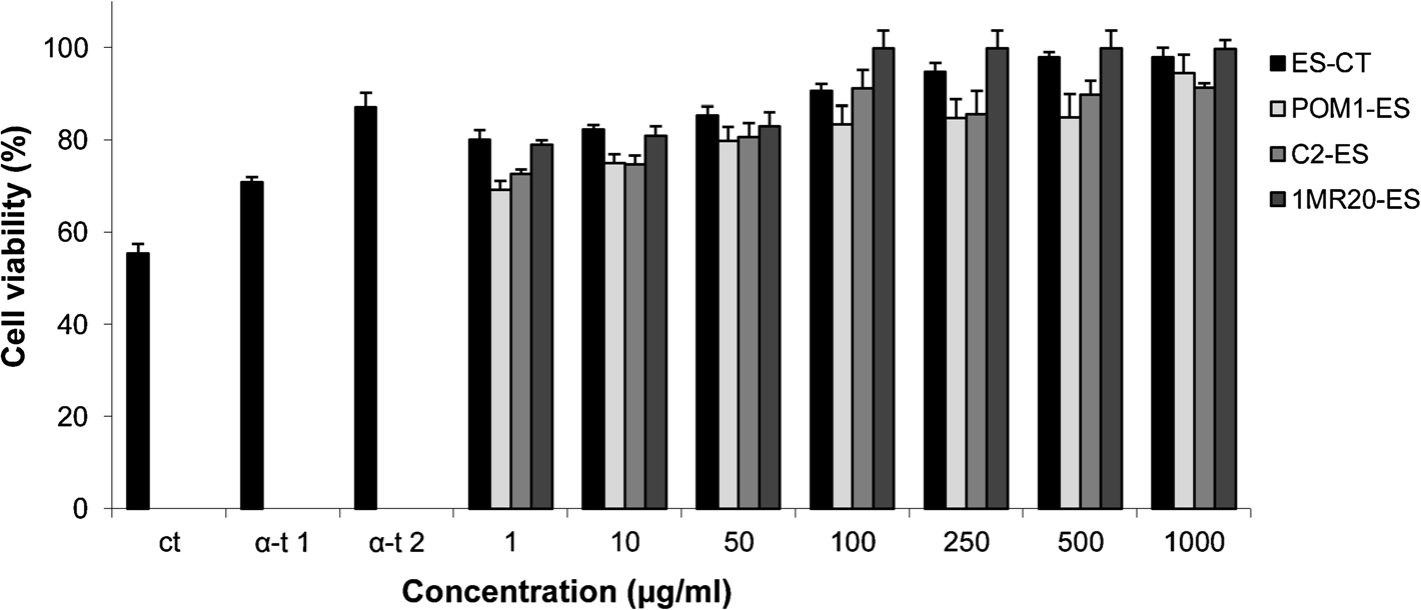
**Cell viability of mouse fibroblasts.** Effect of different concentrations (1 – 1000 μg/ml) of freeze-dried Echinacea suspension (ES) on cell viability of mouse fibroblasts cultured in Dulbecco’s Modified Eagle Medium (DMEM), and incubated with re-suspended freeze dried ES for 16 h. Oxidative stress was artificially induced by incubating cultured cells with 150 μM hydroxide peroxide for 2 h. The percentage of viable cells with respect to untreated cultures was measured through the 3-(4,5-dimethylthiazol-2-yl)-2,5-diphenyltetrazolium bromide (MTT) assay. ES fermented for 24 h at 30°C with *Lactobacillus plantarum* 1MR20 (1MR20-ES), C2 (C2-ES) and POM1 (POM1-ES), and with the association between strains 1MR20 and C2 (1MR20/C2-ES) were assayed. ES-CT, chemically acidified ES, without bacterial inoculum, and incubated for 24 h at 30°C, was the control. α-Tocopherol (α-t1, 250 μg/ml, and α-t2, 500 μg/ml) was used as the positive control. Data are the means of three independent experiments twice analysed.

To further investigate the capacity of ES to act as radical scavenger, cultured mouse fibroblasts were grown in the presence of freeze-dried fermented and non-fermented samples. Afterwards, mouse fibroblasts were treated with hydrogen peroxide. Cell viability was assayed through the capacity of functional mitochondria to catalyze the reduction of MTT to formazan salt via mitochondrial dehydrogenases. Compared to negative control (55.4 ± 2.1% of cell viability after oxidative stress), α-tocopherol and all ES significantly (*P*<0.05) increased cell survival (Figure 1). In particular, ES fermented with *Lb. plantarum* 1MR20, especially at concentrations of 100 and 250 μg/ml, induced the highest cell viability. The activity was also significantly (*P*<0.05) higher than that found with α-tocopherol at both concentrations of 250 and 500 μg/ml. Overall, no significant (*P*>0.05) differences were found among ES-CT and ES fermented with *Lb. plantarum* C2 and POM1.

Based on the above results, *Lb. plantarum* C2 and 1MR20, which, respectively, showed the highest antimicrobial and antioxidant activities were used in association as mixed starter to ferment ES. The antimicrobial and antioxidant activities were confirmed (Tables 1 and 2). Since methanol extractable compounds (e.g., polyphenols), which are responsible for antioxidant activity of *Echinacea* spp., were previously identified [1,30], further characterizations mainly concerned the water-soluble extract.

### Microbiology and chemical characteristics

After 24 h of fermentation, ES fermented with the association of *Lb. plantarum* C2 and 1MR20 had a cell density of presumptive lactic acid bacteria of 7.5 ± 0.4 × 10^9^ CFU/ml. The kinetic of growth was characterized as follows: *A* of 1.3 ± 0.01 log CFU/ml, μ_max_ of 0.11 ± 0.01 log CFU/ml h and λ of 0.21 ± 0.02 h. The pH of the fermented ES was 4.07 ± 0.12 (initial pH of 5.21 ± 0.22). The parameters of the kinetic of acidification were as follows: ΔpH of 1.14 ± 0.03 units, V_max_ of 0.16 ± 0.01 dpH/h and λ of 1.76 ± 0.03 h.

During fermentation, the concentration of glucose and fructose decreased significantly (*P*<0.05) compared to ES-CT (ca. 50 and 25%, respectively) (Table 3). Sucrose was not found. At the end of fermentation, the concentration of lactic and acetic acid was 20 ± 1.2 mM and 3.2 ± 1.8 mM respectively. The concentration of total phenols of ME of ES-CT and fermented ES was 9.4 ± 0.2 and 9.9 ± 0.1 mM, respectively. The concentration of free amino acids of the fermented ES was five times higher than that found for ES-CT. After freeze-drying, the values of moisture, protein, lipids and ash did not significantly (*P*>0.05) vary between ES-CT and fermented ES.

**Table 3:** Chemical characteristics of Echinacea suspension (ES)

	**ES-CT**	**1MR20/C2 -ES**
*Liquid matrix*		
**pH**	4.01 ± 0.04	4.07 ± 0.12
**Glucose (mM)**	10.9 ± 0.5^b^	5.5 ± 0.2^a^
**Fructose (mM)**	11.84 ± 0.6^b^	8.3 ±0.2^a^
**Sucrose (mM)**	-	-
**Total phenols (**mM gallic acid/ml)	9.4 ± 0.2^a^	9.9 ± 0.1^b^
**Lactic acid (mM)**	21.0 ± 2.3	20.0 ± 1.2
**Acetic acid (mM)**	-	3.2 ± 1.8
**Free amino acids (mg/l)**	122 ± 5^a^	612 ± 11^b^
*Freeze dried matrix*		
**Moisture (%)**	2.28 ± 0.15^b^	2.02 ± 0.05^a^
**Protein (% of d.m.)**	15.9 ± 0.3	16.0 ± 0.2
**Lipids (% of d.m.)**	1.31 ± 0.11	1.30 ± 0.23
**Ash (% of d.m.)**	12.78 ± 0.21	12.82 ± 0.19

Data are the mean of three independent analyses.

*d.m*.: dry matter.

^a-b^ Values in the same row with different superscript letters differ significantly (*P<0.05*).

ES was fermented for 24 h at 30°C with the association between *Lactobacillus plantarum* 1MR20 and C2 (1MR20/C2-ES). The chemically acidified ES, without bacterial inoculum, and incubated for 24 h at 30°C (ES-CT), was the control.

### Purification and identification of antimicrobial and antioxidant compounds

Aiming at identifying bioactive compounds, WSE of ES fermented with the association between *Lb. plantarum* 1MR20 and C2 was subjected to ultra-filtration (cut-off 50, 30, 10 and 5 kDa). The antimicrobial activity was assayed using *B. megaterium* F6 as the indicator strain. The antioxidant activity was determined through the DPPH radical scavenging assay. All fractions from ultra-filtration showed both the activities. This suggested that molecular masses of the active compounds were lower than 5 kDa. Therefore, bioactive compounds were present in the last ultra-filtration fraction D. After digestion by trypsin, the antimicrobial activity of fraction D was completely lost. The antioxidant activity decreased from ca. 40 to 25%. This results suggested that the above activities were totally or for the major part related to compounds of protein nature. Both antimicrobial and antioxidant activities were unaffected by heating at 100°C for 5 min.

**Figure 2:**
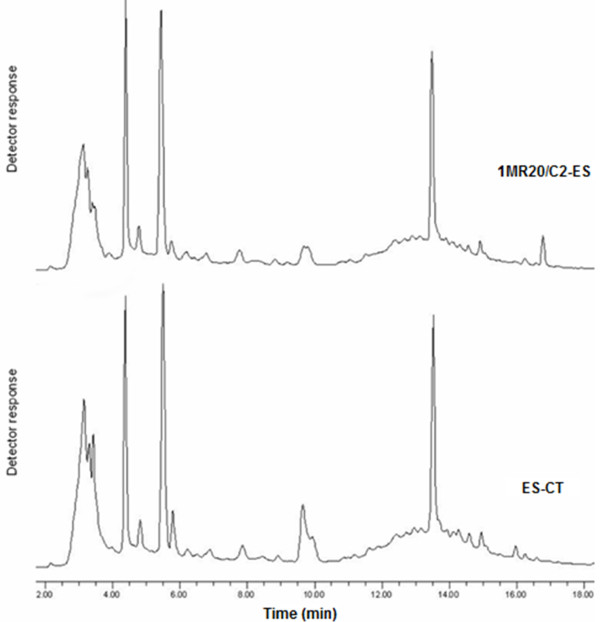
**Polyphenols profiles after High Pressure Liquid Chromatography (HPLC) analysis.** Water-soluble extract (WSE) of Echinacea suspension (ES) fermented for 24 h at 30°C with the association between *Lactobacillus plantarum* 1MR20 and C2 (1MR20/C2-ES). The chemically acidified ES, without bacterial inoculum, and incubated for 24 h at 30°C (ES-CT), was the control.

**Figure 3:**
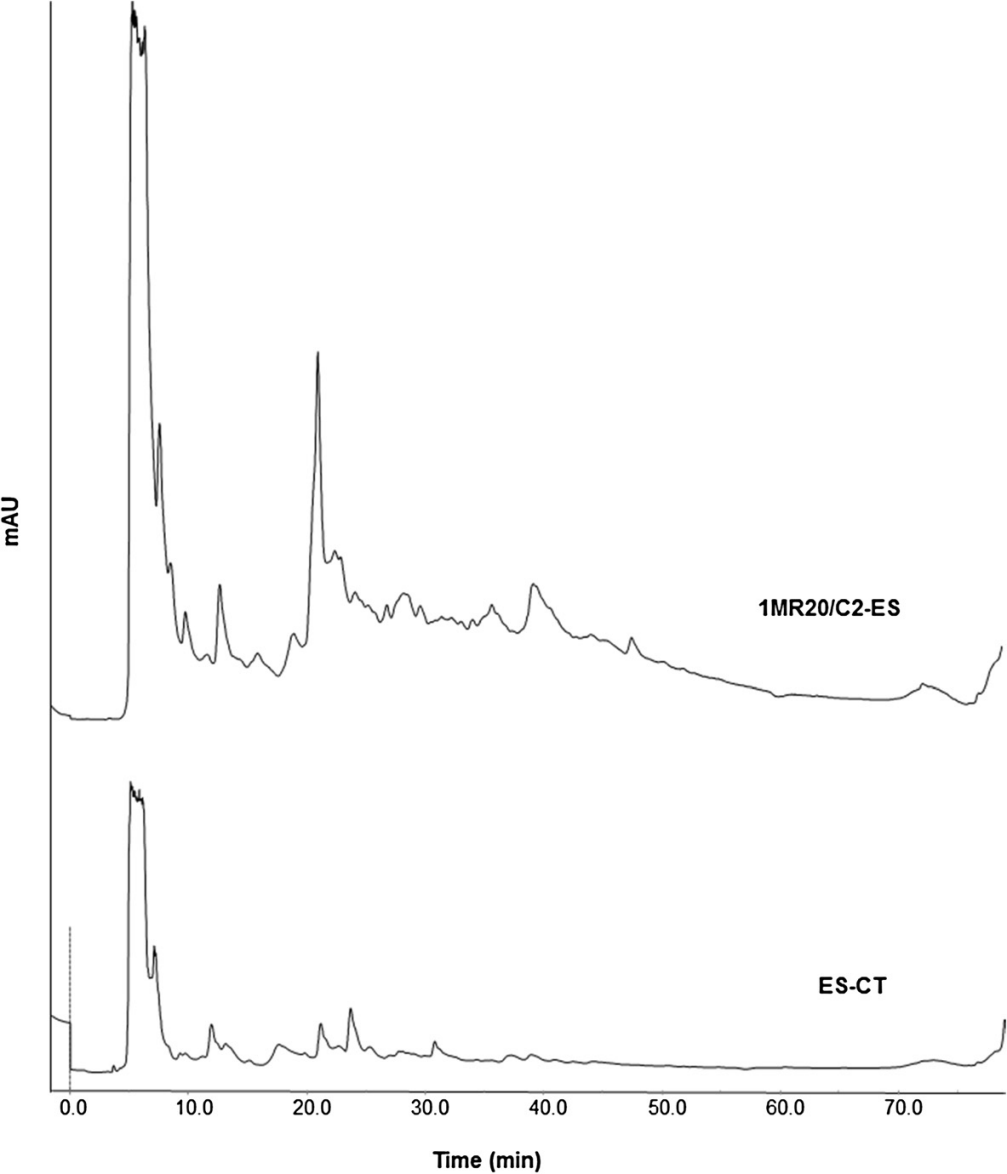
**Peptide profiles after Reverse Phase-Fast Protein Liquid Chromatography (RP-FPLC) analysis.** Water-soluble extract (WSE) of Echinacea suspension (ES) fermented for 24 h at 30°C with the association between *Lactobacillus plantarum* 1MR20 and C2 (1MR20/C2-ES). The chemically acidified ES, without bacterial inoculum, and incubated for 24 h at 30°C (ES-CT), was the control.

As shown through HPLC analysis, the polyphenol profiles of fractions D of WSE from ES-CT and fermented ES were almost similar (Figure 2). Notwithstanding an antioxidant effect due to polyphenols, this probably confirmed that differences between fermented and non-fermented samples had to be attributed also or mainly to compounds of protein nature. As shown through RP-FPLC analysis, a marked increase of several peak areas and higher complexity was found for the peptide profile of fermented ES compared to that of ES-CT (Figure 3). Indeed, the concentration of peptides increased from 2.76 ± 0.31 to 13.91 ± 1.2 mg/ml. As estimated towards *B. megaterium* F6, the MIC of fraction D from fermented ES was 1.4 ± 0.2 mg/ml of peptides.

Aiming at further purifying the antimicrobial and antioxidant compounds, fraction D was subjected to further fractionation through RP-FPLC searching for protein derivatives. Thirty-seven fractions were collected. Antimicrobial activity was only found in fraction 2. On the contrary, the antioxidant activity largely distributed: from fractions 2 to 6 (35-55% of DPPH radical scavenging activity) and from fractions 8 to 23 (45-50% of activity). Fraction 2 was subjected to nano-LC-ESI-MS/MS analysis, which allowed the identification of a mixture of peptides. The most intense peaks corresponded to eight different peptides, which were characterized by sequences containing 7 to 12 amino acid residues (Table 4). Except for MLAAKSSAAST (54%) and VINIVLAAV (88%), the hydrophobic ratio of all peptides was lower than 50%. Except for ENGNTLSG (total net charge -1), neutral or positive total net charges were found for the other peptides. Five peptides were encrypted in the plantaricin H sequence (PlnH). The other peptides derived from proteolysis of plantaricin G (PlnG). Since antioxidant activity of WSE distributed in a very large number of RP-FPLC fractions, no further peptide identification was carried out. Based on the above results, it was hypothesized a non-specific effect of ES protein derivatives, which were liberated through proteolysis during lactic acid bacteria fermentation.

**Table 4:** Antimicrobial peptides

**n.**	**Sequence **^**a**^	**Score**	**Calculated mass**	**Expected mass**	**Delta**	**Source Protein NCBI accession number**
1	TASSVASTTK	49	952.217	952.025	-0.192	ADE34579.1; PlnH, *Lb. plantarum*
2	TIKATKT	38	762.272	761.909	-0.366	ADE34579.1; PlnH, *Lb. plantarum*
3	MLAAKSSAAST	18	1037.511	1037.199	-0.312	ADE08259.1; PlnH *Lb. plantarum*
4	VSSGAEIAKI	15	973.687	974.120	0.433	ADE34579.1; PlnH, *Lb. plantarum*
5	NQMLAAKSSAAS	13	1178.621	1178.329	-0.292	ADE34579.1; PlnH, *Lb. plantarum*
6	VINIVLAAV	46	911.725	911.156	-0.569	ADE34578.1; PlnG, *Lb. plantarum*
7	ENGNTLSG	35	791.11	790.786	-0.324	ADE34578.1; PlnG, *Lb. plantarum*
8	GNMPSGG	12	612.360	612.669	0.339	ADE34578.1; PlnG, *Lb. plantarum*

^a^ The single-letter amino acid code is used.

Sequences of peptides contained in the purified antimicrobial fraction of the water-soluble extract (WSE) of Echinacea suspension (ES) fermented for 24 h at 30°C with the association between *Lactobacillus plantarum* 1MR20 and C2 (1MR20/C2-ES).

### Cytotoxicity and immunmodulatory activities

Preliminarily, the citoxicity of freeze dried ES fermented with *Lb. plantarum* POM1, 1MR20 and C2 were assayed towards human colon adenocarcinoma Caco-2 cells. The proliferation of Caco-2 cells was determined through MTT assay. Cells were plated at low density (5 × 10^4^ cells per well) into EMEM, without phenol red, and treated with various concentrations (1-500 μg/ml) of ES for 24, 48 and 72 h. Compared to basal serum free medium (control), incubation with 1-500 μg/ml of fermented ES did not significantly (*P* >0.05) affect cell proliferation (data not shown). The same result was found after treatment with ES-CT.

**Figure 4:**
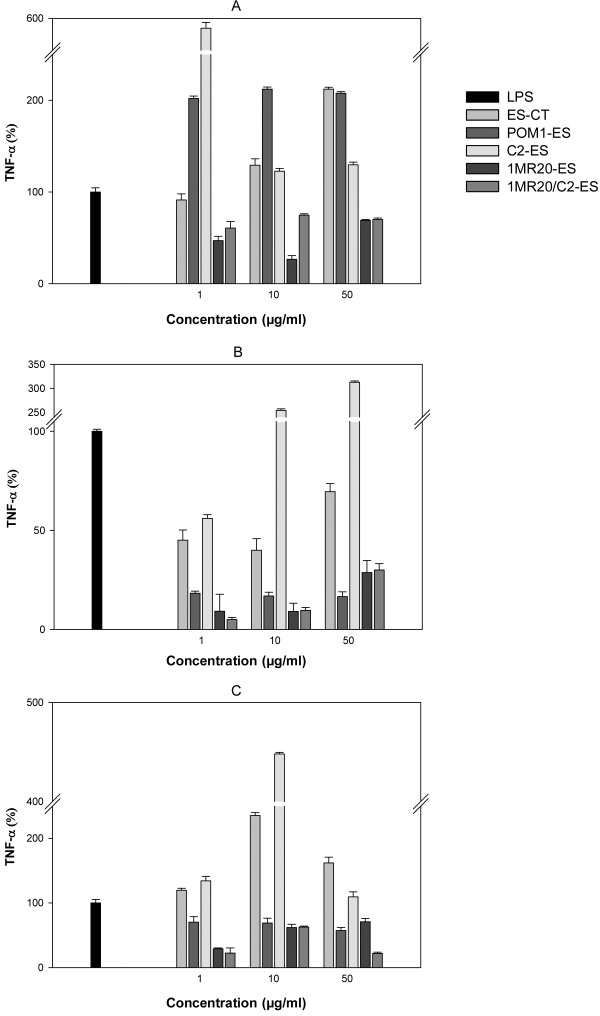
**Expression of TNF-α gene of Caco-2 cells.** The expression was analyzed after treatment with Echinacea suspension (ES) (1-50 μg/ml) for 16 (**A**), 24 (**B**), and 48 h (**C**), as determined by RT-PCR. ES fermented for 24 h at 30°C with *Lactobacillus plantarum* 1MR20 (1MR20-ES), C2 (C2-ES) and POM1 (POM1-ES), and with the association between strains 1MR20 and C2 (1MR20/C2-ES) were assayed. ES-CT, chemically acidified ES, without bacterial inoculum, and incubated for 24 h at 30°C, was the control. Results were expressed as percent ratio to lipopolysaccharide (5 μg/ml) (LPS)-treated cells (LPS). Data are the means of three independent experiments twice analysed.

Regarding immune-modulatory activity, the treatment with fermented ES (1, 10 and 50 μg/ml) exerted a modulatory effect on Caco-2 cells (Figure 4A, B and C). The significance of this effect was evaluated in comparison with the treatment of Caco-2 cells by basal medium, which contained containing LPS (5 μg/ml) (control). The exposition for 16 h to ES fermented with *Lb. plantarum* 1MR20significantly (*P*<0.05) decreased the expression of *TNF*-α gene (Figure 4A). The activity was significantly (*P*<0.05) higher than that of ES-CT. After 24 and 48 h, the highest immune-modulatory activity was found for ES fermented with 1MR20 (1 μg/ml) (Figure 4B and 4C). The ES fermented with the association between the two strains 1MR20 and C2-ES had an effect similar to the ES fermented with strain 1MR20 alone Nevertheless, the effect was higher when the concentration of 50 μg/ml was assayed for 48 h of incubation.

## Discussion

Health benefits from fermented plant materials are usually expressed either directly, through interaction of ingested live microorganisms with the host (probiotic effect), or indirectly as the result of the ingestion of microbial metabolites, which are synthesized during fermentation (biogenic effect) [31]. To the best of our knowledge, this is the first study reporting the capacity of lactic acid bacteria to release bioactive compounds during fermentation of *Echinacea* spp.

Under optimal processing conditions, microorganisms may contribute to plant functionality through their enzyme portfolio, which promotes the release of various metabolites, such as free amino acids and vitamins [31]. Bioactive peptides and γ-amino butyric acid (GABA) may be released during food processing at levels higher than the physiological threshold, thus exerting various *in vivo* health benefits [31].

Besides well-known positive effects during food fermentation (e.g., dairy products, fermented meats and leavened baked goods), lactic acid bacteria are recently used to synthesize GABA from grape must [25], isoflavone, aglycones and equol from soy milk, antioxidant and anti-hypertensive peptides, and lunasin from various cereal and pseudo-cereal flours [26,32,33]. Strains of *Lactobacillus plantarum*, previously isolated from vegetable matrices that are particularly rich of polyphenol compounds [34-36], were used as starters. The concentration of fermentable carbohydrates of Echinacea powder was enough to allow bacterial growth but to get optimal fermentation performances, yeast extract was added. The concentration of fermentable carbohydrates of Echinacea powder was enough to allow bacterial growth.

Antimicrobial and antioxidant activities were investigated during fermentation and compared to non-fermented Echinacea suspension. Echinacea suspensions fermented with *Lb. plantarum* POM1 and, especially, strain C2 exhibited a marked antimicrobial activity towards Gram-positive and -negative bacteria,while it was very low towards fungi. According to literature data [37-40], the concentration of polyphenol compounds largely varies among commercial products containing *Echinacea* spp. Genetic variation and environmental inputs (e.g., light, temperature, agronomic practices) are considered the main factors affecting the levels of caffeic acid and its derivatives. Drying temperature, extraction methods, formulation and storage conditions may also be responsible for this variability. Overall, the radical scavenging activity of polyphenols is mainly influenced by the number of hydroxyl groups of the aromatic ring [41,42]. The radical scavenging activity of the methanol extracts of Echinacea suspensions was high and almost similar between fermented and non-fermented preparations. The only exception was represented by fermentation with *Lb. plantarum* 1MR20, which also caused an increase of antioxidant activity of the methanol extract. A marked inhibition of the linoleic acid peroxidation was also found during eight days of incubation. Overall, this antioxidant potential had to be mainly attributed to inherent polyphenols from the Echinacea matrix, which are solubilized with ethanol or methanol [1]. On the contrary, the antioxidant activity of water-soluble extracts was markedly affected by lactic acid bacteria fermentation. When *Lb. plantarum* 1MR20 was used as starter, the DPPH radical scavenging activity was at least twice than that of the non-fermented Echinacea suspension. MTT assays on mouse fibroblasts showed that the protective effect towards induced oxidative stress by freeze dried Echinacea suspension fermented with *Lb. plantarum* 1MR20 was higher than that of nonfermented sample and also higher than that of α-tocopherol, which was used at the same concentration.

Aiming at combining antimicrobial and antioxidant activities, *Lb. plantarum* C2 and 1MR20 were used in association to ferment the Echinacea suspension. The fermentation favored the liberation of high levels of free amino acids and peptides, which increased ca. 5-6 times compared to non-fermented suspension. The protocol used for purification demonstrated that compounds responsible for antimicrobial activity had low molecular masses and were affected by treatment with digestive enzymes. A mixture of peptides, having 7 to 12 amino acid residues, was identified. All the sequences were encrypted in plantaricins PlnH or PlnG, which are expressed via the conserved part of the bacteriocin *loci* and which are responsible for antimicrobial activities of *Lb. plantarum* strains [43]. The molecular mechanism via quorum sensing regulation of constitutive plantaricin synthesis by *Lb. plantarum* C2 was recently described [44]. Although the functions of PlnH and PlnG are already described in the literature [43], no information is available on the activity of their hydrolysis products. In some cases, the bioactivity of hydrolysis products is higher than that of the native precursor [27,45]. The synergistic activity of a mixture of peptides is frequently expected [27,45]. The MIC of the purified fraction, which contained the mixture of the identified peptides, was comparable to the values reported for similar compounds [46].

During RP-FPLC purification, the antioxidant activity of the water-soluble extract from Echinacea suspensions was distributed in a large number of fractions, and was proportional to the peptide concentration. The contribution of various bioactive compounds was hypothesized. Since polyphenols show variable solubility in water and organic solvents [1], a contribution from water-soluble polyphenols, was not excluded. Nevertheless, a conspicuous part of the antioxidant activity was lost through treatment with digestive enzymes. Fermentation promoted a marked increase of peptides concentration, which were distributed throughout the acetonitrile gradient. Previously, several peptides were shown to possess antioxidant capacity [45]. Antioxidant peptides from vegetable matrices are considered to be safe and healthy compounds with low molecular weight, low cost, high activity and easy absorption. Compared to antioxidant enzymes, peptides have higher stability under various environmental conditions and no hazardous immunoreaction due to their simpler structure [45]. The use of protein hydrolysates obtained through fermentation has been already proposed for nutraceutical purposes [45]. Non purified protein hydrolysates have certain benefits over purified peptides. The absorption of peptides, in fact, increases in the presence of sugars and amino acids, and as the consequence, also the antioxidant activity [45]. The exact mechanism underlying the antioxidant activity of peptides is not fully understood. It was hypothesized that they act as inhibitors of lipid peroxidation [45], scavengers of free radicals [45] and chelators of transition metal ions [45]. Antioxidant peptides may protect cells from damage by Reacting Oxygen Species (ROS) through gene induction.

MTT assay on Caco-2 cells demonstrated the absence of cytotoxicity for a wide range of concentrations of Echinacea suspension. The immune-modulatory effect of Echinacea extracts was proven through *in vitro* and *in vivo* assays [10]. It might be associated with various compounds such as polysaccharydes, alkylamides and caffeic acid derivatives. Fermentation of Echinacea suspension, especially with *Lb. plantarum* 1MR20, markedly affected the expression of TNF-α by Caco-2 cells. The *in vitro* assay was carried out using LPS from *E. coli*, which represents the principal shock inducing factor from the outer membrane of Gram-negative bacteria. Pro-inflammatory cytokines derived from LPS-stimulated cells are responsible for lethal effects as well as for the generation of intermediate signals, which amplify the cellular response by triggering the production of chemokines [47]. TNF-α is a pleiotropic inflammatory cytokine, which mediates inflammation, immune-response and apoptosis [48]. A large spectrum of diseases involved the over production or the persistent activation of TNF-α [49]. This cytokine possesses both growth stimulating and inhibitory properties, and its release is self-regulatory. For instance, TNF-α induces neutrophil proliferation during inflammation and also neutrophil apoptosis upon binding the TNF-R55 receptor [50]. Low levels of TNF-α may contribute to homeostasis by regulating the body circadian rhythm [51].

## Conclusions

Nutraceutical industry and preventive medicine are currently showing a marked interest for natural antimicrobial, antioxidant and immune-modulatory compounds. The demand for dietary phytonutrients encourages the exploitation of plant potential through lactic acid fermentation [1]. Under this perspective, this study demonstrates how the antimicrobial, antioxidant and immune-modulatory properties of *Echinacea* spp. are enhanced through the fermentation by selected lactic acid bacteria. Novel applications as functional food dietary supplements or pharmaceutical preparations could be expected.

## Methods

### Microorganisms and culture conditions

*Lactobacillus plantarum* POM1, 1MR20 and C2 were previously isolated, identified by partial sequencing of 16S rRNA, recA, pheS and rpoA genes, and selected from tomato, pineapple and carrot, respectively [34-36]. Strains were cultivated for 24 h at 30°C on MRS broth (Oxoid, Basingstoke, Hampshire, United Kingdom). When used for fermentation, lactic acid bacteria cells were cultivated until the late exponential phase of growth was reached (ca. 10 h), washed twice in 50 mM phosphate buffer, pH 7.0, and re-suspended in the liquid substrate. Enumeration of lactic acid bacteria was carried out by plating onto MRS agar at 30°C for 48 h.

### Fermentation media

The commercial powder of *Echinacea purpurea* (L.) Moench (Epo S.r.l., Milano, Italy) was used as the substrate for fermentation. Echinacea powder suspension (ES) (5%, wt/vol) was made in (i) distilled water; (ii) distilled water, which contained 0.4% (wt/vol) yeast extract (Oxoid); or (iii) grape must (1% of total carbohydrates). ES was sterilized at 121°C for 15 min and inoculated with lactic acid bacteria strains at the initial cell density of ca. 1 × 10^8^ CFU/ml. As preliminarily shown, lower inoculums did not ensure the same functional activities (data not shown). Fermentation was allowed at 30°C for 24 h, under stirring conditions (120 rpm). ES, without bacterial inoculum, and chemically acidified with lactic acid (final pH 4.0), was incubated under the same conditions and used as the control (ES-CT).

### Kinetics of growth and acidification

Kinetics of growth and acidification were determined and modelled in agreement with the Gompertz equation, as modified by Zwietering et al. [52]: *y*= *k* + A exp{- exp[(*μ*_max_ or V_max_ e/A)(*λ*-t) + 1]}; where *y* is the growth expressed as log CFU/g/h or the acidification rate expressed as dpH/dt (units of pH/h) at the time *t*; *k* is the initial level of the dependent variable to be modelled (log CFU/g or pH units); *A* is the cell density or pH (units) variation (between inoculation and the stationary phase); *μ*_max_ or V_max_ is the maximum growth rate expressed as Δ log CFU/ml h or the maximum acidification rate expressed as dpH/h, respectively; *λ* is the length of the lag phase measured in hours. The experimental data were modelled by the non-linear regression procedure of the Statistica 8.0 software (Statsoft, Tulsa, USA).

### Characterization of Echinacea powder suspension (ES)

Aliquots (1 ml) of ES were centrifuged at 10,000 × g for 10 min. The supernatant (water-soluble extract, WSE) was filtered through a Millex-HA 0.22-mm pore size filter (Millipore Co., Bedford, MA). The concentration of glucose, fructose and sucrose was determined through HPLC analysis, using an ÄKTA Purifier system (GE Healthcare) equipped with a Spherisorb column (Waters, Millford, USA) and a Perkin Elmer 200a refractive index detector. Elution was at 32°C with a flow rate of 1 ml/min, using acetonitrile 80% as the mobile phase [53].

Organic acids were determined by HPLC, using an ÄKTA Purifier system (GE Healthcare) equipped with an Aminex HPX-87H column (ion exclusion, Biorad) and a UV detector operating at 210 nm. Elution was at 60°C with a flow rate of 0.6 ml/min, using 10 mM H_2_SO_4_ as the mobile phase [54]. Peaks were identified by comparing elution times and spiking samples with known quantities of standard solutions of acetic and lactic acid. Total and individual free amino acids were analysed by a Biochrom 30 series Amino Acid Analyzer (Biochrom Ltd., Cambridge Science Park, England), with a Na-cation-exchange column (20 by 0.46 cm inner diameter) as described by Rizzello et al. [55].

To determine the gross composition samples were previously freeze-dried. Dry matter was assessed by an infrared moisture analyzer MAC 110/NP (Radwag, Poland). Total nitrogen was measured by the Kjeldahl method no. 920.87 [38], using a VELP DK6 heating digester and a VELP UDK 130 distillation system (VELP Scientifica, Usmate Milano). Ash was determined by gravimetric method AOAC no. 923.03 [38]. The fat content was measured by solvent extraction, using a Soxhlet extraction unit.

### DPPH radical scavenging activity

The 1,1-diphenyl-2-picrylhydrazyl (DPPH) radical scavenging activity of ES was determined both on methanol extract (ME) and WSE. Five milliliters of ES were mixed with 50 ml of 80% methanol to get ME. The mixture was purged with nitrogen stream for 30 min, under stirring condition, and centrifuged at 4,600 × *g* for 20 min. ME were transferred into test tubes, purged with nitrogen stream and stored at ca. 4°C before analysis. The concentration of total phenols was determined as described by Slinkard and Singleton [56]. It was expressed as gallic acid equivalent. The free radical scavenging capacity was determined using the stable 2,2-diphenyl-1-picrylhydrazyl radical (DPPH˙), as reported by Yu et al. [57]. The reaction was monitored by reading the absorbance at 517 nm every 2 min for 30 min. A blank reagent was used to verify the stability of DPPH˙ over the test time. The absorbance value measured after 10 min was used for the calculation of the μmoles DPPH˙ scavenged by extracts. The absorbance value in the presence of the extract was also determined over 30 min and compared with 75 ppm butylated hydroxytoluene (BHT) as the antioxidant reference.

The scavenging effect of freeze dried WSE on DPPH˙ free radical was measured according to the method of Shimada et al. [58], with some modifications. Freeze-dried samples were first dissolved in 0.1 M phosphate buffer pH 7.0 at the final concentration of 1 mg/ml of peptides, and then 2 ml were added to 2 ml of 0.1 mM DPPH, which was dissolved in 95% ethanol. The mixture was shaken and left at room temperature for 30 min. The absorbance was read at 517 nm. The absorbance measured after 10 min was used for the calculation of the DPPH scavenged by WSE [53]. More the absorbance was low, higher it was the DPPH scavenging activity. The scavenging activity was expressed as follows: DPPH scavenging activity (%) = [(blank absorbance – sample absorbance) / blank absorbance] × 100. BHT (1 mg/ml) was used as the antioxidant reference.

### Inhibition of linoleic acid autoxidation

The antioxidant activity of ME and WSE was also measured according to the method of Osawa and Namiki [59], with some modifications. Freeze dried WSE or ME (1 mg) was suspended into 1 ml of 0.1 M phosphate buffer (pH 7.0), and added to 1 ml of linoleic acid (50 mM), previously dissolved on ethanol (99.5%). Incubation in glass test tube, tightly sealed with silicone rubber cap, was allowed at 60°C in the dark for 8 days. The degree of oxidation was determined by measuring the value of ferric thiocyanate, according to Mitsuta et al. [60]. One hundred microliters of the above sample were mixed with 4.7 ml of 75% (v/v) ethanol, 0.1 ml of 30% (w/v) ammonium thiocyanate and 0.1 ml of 0.02 M ferrous chloride, dissolved in 1 M HCl. After 3 min, the color development (degree of linoleic acid oxidation) was measured spectrophotometrically at 500 nm. BHT and α-tocopherol (1 mg/ml) were used as the antioxidant references. A negative control (without antioxidant) was also considered. The inhibition effect was expressed as follows: inhibition of linoleic acid autoxidation (%) = [(negative control absorbance – sample absorbance) / negative control absorbance] × 100.

### Viability of oxidation-induced cells

Mouse fibroblasts (Balb 3T3, clone A31, ATCC CCL-163TM) were cultured under humidified atmosphere (5% CO_2_, 37°C), using Dulbecco’s Modified Eagle Medium (DMEM), which was supplemented with 10% (w/v) calf bovine serum (CBS), 1% penicillin (10,000 U/mL)/streptomycin (10,000 U/mL) mixture, and 1% non-essential amino acid solution (NEAA). The culture medium was renewed every two days and after four passages the cultures were used for viability assays. Cell viability was measured using the MTT (3-(4,5-dimethyl-2-yl)-2,5-diphenyltetrazolium bromide) method [61]. The capacity of succinate dehydrogenase to convert 3-(4,5-dimethylthiazol-2-yl)-2,5-diphenyltetrazolium bromide into visible formazan crystals was assessed. For MTT assay, cells were seeded into 96-well plate (Becton Dickinson France S.A., Meylan Cedex, France) at the density of 5 × 10^4^ cells/well and incubated for 24 h. Subsequently, cells were treated with re-suspended freeze dried ES and incubated for further 16 h. The concentration of ES in the reaction mixture varied from 1, 10, 50, 100, 250, 500 and 1000 μg/ml. A negative control, without addition of ES, was used. α-Tocopherol (250 and 500 μg/ml ) was used as the positive control. Following removal of CBS, cells were exposed to 150 μM hydrogen peroxide for 2 h. For each well, 100 μl of MTT (0.5 mg/ml final concentration), dissolved in DMEM, were added and incubated (37°C, 5% CO_2_) in the dark for 3 h. Finally, 100 μl of dimethyl sulphoxide (DMSO) were added to dissolve purple formazan product. The solution was shacked in the dark for 15 min at room temperature. The absorbance of the solution was read at 570 nm in a microplate reader (BioTek Instruments Inc., Bad Friedrichshall,Germany). Each experiment was carried out in triplicate. Data were expressed as the mean percentage of viable cells compared to the control culture, without oxidative stress.

### Antimicrobial activity

*Escherichia coli* DSM 30083 and *Enterobacter aerogenes* DSM 30053 grown on Luria Bertani (LB) broth (Oxoid) at 37°C, *Enterococcus durans* DSM 20633 and *Yersinia enterocolitica* DSM 4780 grown on Brain Hearth Infusion (BHI) (Oxoid) at 37°C, *Weissella confusa* DSM 20196 and *Leuconostoc lactis* DSM 20202 grown on MRS broth (Oxoid) at 30°C, and *Propionibacterium jensenii* DSM 20535 grown in sodium-lactate broth at 37°C, which belong to the Culture Collection of the Leibniz Institute DSMZ (Braunschweig, Germany), and *Lactobacillus sakei* SAL1 grown on MRS broth at 30°C, *Bacillus megaterium* F6 grown on LB broth at 37°C, *Candida krusei* DSM 3433 grown on Yeast Extract Peptone Dextrose (YEPD) broth (Oxoid) at 25°C, and *Penicillium roqueforti* DPPMAF1 grown on Potato Dextrose Agar (PDA) (Oxoid) at 25°C, which belong to the Culture Collection of the Department of Soil, Plant and Food Sciences (University of Bari, Italy), were used to assay the antimicrobial activity.

The well-diffusion assay [62] was used to determine the antimicrobial activity of WSE and further partially purified fractions. For *P. roqueforti* DPPMAF1, the hyphal radial growth inhibition assay was used, as previously described by Coda et al. [63]. After this preliminary assay, the antimicrobial activity of WSE fractions was determined through the broth micro-dilution technique [64]. Only bacteria were considered for this assay. Logarithmic phase cells (ca. 10^8^ CFU/ml) were harvested by centrifugation (8000 × g for 10 min), washed twice with 10 mM phosphate buffer, pH 7.0, and adjusted to 10^4^ CFU/ml. The sterile 96-well microtiter plate was used (Greiner Labortechnik). Fifty microliters of each cell suspension were mixed with 50 μl of each fraction, and 100 μl of each specific culture broth were added. The estimated peptide concentration of each fraction ranged from 5 to 2000 μg/ml. Control wells contained all the components except for the peptide fraction, which was replaced with distilled water (positive control) or with chloramphenicol (100 μg/ml) (negative control). Microplates were incubated at 25, 30 or 37°C depending on the strain, and growth was monitored over 24 h by measuring the optical density of the culture at 620 nm using a microplate reader (Dynatech Laboratories, West Sussex, UK). The Minimum Inhibitory Concentration (MIC) corresponded to the lowest concentration of the peptide fraction needed to fully inhibit the bacterial growth. When growth was inhibited, cells were recovered from microplates, washed twice with 10 mM phosphate buffer, pH 7.0, and incubated into fresh medium to allow the recovery of growth. All assays were carried out in triplicate.

### Purification of antioxidant and antimicrobial compounds

WSE (ca. 400 μl) was subjected to fractionation by ultra-filtration (Ultrafree-MC centrifugal filter units, Millipore), using membrane sizes of 50, 30, 10 and 5 kDa cut-off (corresponding to fractions A, B, C and D, respectively). Centrifugation was at 10,000 *× g* for 60 min.

Polyphenols were analyzed using a multi-solvents delivery system controller 600 (Waters, PA, USA), equipped with a PDA 996 (Waters) and a Synergi Hydro 80Ǻ column, 5 μm particle size, 250 × 4.6 mm (Phenomenex, CA, USA). Separation was carried out using a binary gradient of 10% (v/v) formic acid in water (solvent A) and acetonitrile (CH_3_CN) (solvent B). The initial conditions were flow 0.8 ml/min, column temperature 30°C and solvent B 12%; after 1 min, the concentration of B was linearly increased to 90% in 35 min. The absorbance between 210 and 400 nm was acquired.

Peptides were analyzed by Reversed-Phase Fast Performance Liquid Chromatography (RP-FPLC), using a Resource RPC column and an ÄKTA FPLC equipment, with the UV detector operating at 214 nm (GE Healthcare Bio-Sciences AB, Uppsala, Sweden). Aliquots of WSE, containing ca. 1 mg/ml of peptides, were added to 0.05% (v/v) trifluoroacetic acid (TFA) and centrifuged at 10,000 × *g* for 10 min. The supernatant was filtered with a 0.22 μm pore size filter and loaded onto the column. Gradient elution was carried out at the flow rate of 1 ml/min, using a mobile phase composed of water and CH_3_CN, containing 0.05% TFA. The concentration of CH_3_CN was increased linearly from 5 to 46% between 16 and 62 min, and from 46 to 100% between 62 and 72 min. Solvents were removed from collected fractions by freeze drying. The fractions were re-dissolved in sterile water and subjected to assays for antioxidant and antimicrobial activities.

### Proteolysis and heat stability of partially purified fractions

Partially purified fractions from WSE, which had the highest antimicrobial activity, were subjected to sequential protein hydrolysis by digestive enzymes, according to the method of Pasini et al. [65]. Freeze dried WSE, containing ca. 10 mg of peptides, was suspended into 400 μl of 0.2 N HCl (pH 2.0), containing 0.05 mg/ml of pepsin (EC 3.4.23.1) (Sigma Aldrich CO., St. Louis, MO), and homogenized with a Sterilmixer Lab (PBI International). After 30 min of incubation at 37°C under stirring conditions (150 rpm), 115 μl of 1 M boric acid and 0.5 N NaOH, adjusted to pH 6.8 with 5 N HCl, which contained 0.25 mg/ml of pancreatin (Sigma) and 0.0087 mg/ml of trypsin (EC 3.4.21.4) (Sigma), were added. The resulting pH was 7.6. Pancreatic digestion was lasting 150 min. Digested sample was heated for 5 min at 100°C and centrifuged at 12,000 × g for 20 min, to recover the supernatant. After treatments, the assays for antimicrobial and antioxidant activities were carried out.

### Identification of antimicrobial peptides

Fractions of WSE with the highest antimicrobial activity were subjected to further purification through RP-HPLC, using an ÄKTA Purifier apparatus (GE HealthcareHealthcare Bio-Sciences Corp., Piscataway, New Jersey, USA). The centers of the peaks were collected, freeze dried and used for mass spectrometry analysis. Identification of peptides was carried out by nano-Liquid Cromatography-Electrospray Ionisation-Mass Spectra/Mass Spectra (nano-LC-ESI-MS/MS), using a Finningan LCQ Deca XP Max ion trap mass spectrometer (ThermoElectron) through the nano-ESI interface. According to manufacturer's instrument settings for nano-LC-ESI-MSMS analyses, MS spectra were automatically taken by Xcalibur software (ThermoElectron), in positive ion mode. MS/MS spectra were processed using the software BioWorks 3.2 (ThermoElectron) generating peaklists suitable for database searches. Peptides were identified using MS/MS ion search of Mascot search engine (Matrix Science, London, England) and NCBInr protein database (National Centre for Biotechnology Information, Bethesda, USA). For identification of peptides the following parameters were considered: enzyme: "none"; instrument type: "ESI-trap"; peptide mass tolerance: ± 0.1% and fragment mass tolerance: ± 0.5 Da. Results from peptide identification were subjected to a manual evaluation, as described by Chen et al. [66], and the validated peptide sequences explained all the major peaks in the MS/MS spectrum.

### Cell viability of human colon adenocarcinoma Caco-2 cells

Human colon adenocarcinoma Caco-2 cells (ICLC HTL97023) supplied by the National Institute for Cancer Research (Genoa, Italy) were routinely cultured in Eagle’s minimum essential medium (EMEM), with Earle's balanced salt solution (EMEM-EBSS), and supplemented with 10% (wt/vol) heat inactivated fetal bovine serum (FBS), 1% (wt/vol) NEAA, penicillin (10,000 U/ml)/streptomycin (10,000 μg/ml) and 1% L-glutamin (basal medium). Cells were maintained in 25 cm^2^ culture flasks at 37°C with 5% CO_2_. The culture medium was replaced three times a week. Cell viability was measured according to the MTT assay (see elsewhere). After 24 h seeding on 96 well plate, 80% confluent Caco-2 cells were exposed to various concentrations (1, 10, 50, 100, 250 and 500 μg/ml) of freeze dried ES. The control was the basal medium. Plates were incubated at 37°C, 5% CO_2_, for 24, 48 and 72 h. After each treatment, the medium was aspirated and replaced with 100 μl per well of MTT solution. MTT was dissolved (5 mg/ml) in FBS and diluted 1:10 in the cell culture medium without phenol red. After 3 h of incubation, the basal medium was aspirated and 100 μl per well of DMSO were added to dissolve purple formazan product. The solution was shacked in the dark for 15 min at room temperature. The absorbance of the solutions was read at 570 nm in a microplate reader (BioTek Instruments Inc., Bad Friedrichshall, Germany). Each experiment was carried out in triplicate. Data were expressed as the mean percentage of viable cells compared to the culture in basal medium.

### RNA extraction and real-time-PCR

After treatment with ES, the expression of *TNF-α* from Caco-2 cells was investigated through RT-PCR. When ca. 80% confluence was reached, Caco-2 cells were harvested with trypsin/EDTA, seeded, at the density of 1 × 10^6^ cells per well, into 12-well (Becton Dickinson France S.A., Meylan Cedex, France) plates and incubated at 37°C, 5% CO_2_, for 24 h. Cells in EMEM medium and EMEM with lipopolysaccharide (LPS) (5 μg/ml) were used as the controls. Freeze dried ES at the concentrations of 1, 10, and 50 μg/ml was added to 80% confluent Caco-2 cells with LPS (5 μg/ml), and incubated at 37°C for 16, 24 and 48 h. For quantitative real-time PCR (RT-PCR), total RNA from Caco-2 cells was extracted using Tri Reagent (Sigma Aldrich), as described by Chomczynski and Mackey [67]. The cDNA was synthesized from 2 μg RNA template in a 20 μl reaction volume, using the High-Capacity cDNA Reverse Transcription Kit (Applied Biosystems, Monza, Italy). Ten microliters of total RNA were added to the Master Mix and subjected to reverse transcription in a thermal cycler (Stratagene Mx3000P Real Time PCR System, Agilent Technologies Italia S.p.A., Milan, Italy). The conditions were as follows: 25°C for 10 min, 37°C for 120 min and 85°C for 60 s. The cDNA was amplified and detected through TaqMan® assay (Applied Biosystems). Hs00174128_m1 (*TNF-* α) and Hs999999_m1 (human glyceraldehyde-3-phosphate dehydrogenase, *GAPDH*) were used for Taqman gene expression assays. Human *GAPDH* was the housekeeping gene. PCR amplifications were carried out using 40 ng of cDNA on a 20 μl of total volume. The mixture reaction contained 10 μl of 2× TaqMan Universal PCR Master Mix, 1 μl of 20× TaqMan gene expression assay, 5 μl of water and 4 μl of cDNA. PCR conditions were as follows: 50°C for 2 min (for optimal AmpErase® UNG activity) and 95°C for 10 min, followed by 40 amplification cycles (95°C for 15 s; 60°C for 1 min). Analyses were carried out in triplicate. The average value of target gene was normalized using *GAPDH* gene and the relative quantification of the levels of gene expression was determined by comparing the Δ cycle threshold (ΔCt) value [68]. Results were expressed as percent ratio to LPS-treated cells.

### Statistical analysis

Data were subjected to one-way ANOVA; pair-comparison of treatment means was achieved by Tukey’s procedure at *P*<0.05, using the statistical software, Statistica for Windows (Statistica7.0 per Windows). Student’s t-test was used for MTT assay (GraphPAD 6.0 per Windows).

## Competing interest

The authors declare that they have no competing interests.

## Authors' contributions

CGR carried out purification and identification of bioactive compounds and elaboration of the data; RC carried out the experimental design of the work, microbiological analyses, and elaboration of the data; DSM, and PF performed fermentations and microbiological determinations; DP, BM, and GG carried out ex-vivo assays and related experimental procedures; VMP carried out the chemical analyses; RDC and MG were the supervisors and the coordinators of the research units. All authors read and approved the final manuscript.
